# Prosthetic joint infection due to *Salmonella*species: a case series

**DOI:** 10.1186/s12879-014-0633-x

**Published:** 2014-11-26

**Authors:** Arjun Gupta, Elie F Berbari, Douglas R Osmon, Abinash Virk

**Affiliations:** Division of Infectious Diseases, Department of Medicine, Mayo Clinic, 200 1st Street SW, Rochester, 55905 MN USA; Department of Internal Medicine, UT Southwestern Medical Center, Dallas, Texas USA

**Keywords:** Salmonella, Prosthetic joint infections, Arthroplasty

## Abstract

**Background:**

Prosthetic joint infection (PJI) due to *Salmonella* is rare. Numerous outbreaks of *Salmonella* have been reported throughout the United States in the last decade. We reviewed and analyzed cases of *Salmonella* PJI seen at our institution.

**Methods:**

The medical records of all patients diagnosed with a *Salmonella* PJI between 1969–2013 were reviewed. Patients were followed till death, treatment failure or loss to follow-up.

**Results:**

Six patients of *Salmonella* PJI were identified during the 44 year study period. Five were male; median age was 63.5 years (range 52–76). Five patients were immunodeficient. Five had a total hip arthroplasty infection, while one had a total knee arthroplasty infection. Median prosthesis age at the time of diagnosis of first episode of *Salmonella* PJI was 5 years (range 4 months-9 years). Four presented with fever and constitutional signs within two weeks of symptom onset. Two patients each had gastrointestinal symptoms and *Salmonella* bacteremia. *Salmonella enterica* serovar Enteritidis was the most common organism isolated (4 patients). None were *Salmonella enterica* serovar Typhi. Initial management included aspiration and antimicrobial therapy only (3), debridement and component retention (1) and two-staged exchange (2). All four patients treated without resection failed treatment a median of 2.5 months (range 2–11) after diagnosis and required resection arthroplasty. All six patients who underwent prosthesis removal (and exchange or arthrodesis) had successful outcome with a median duration of follow-up of 11 years (range 4–21). Three of these received oral antimicrobial therapy for a median duration eight weeks (range 4–8) and three received parenteral antimicrobial therapy for a median duration of six weeks (range 4–6).

**Conclusions:**

The increase in *Salmonella* outbreaks does not seem to lead to increased *Salmonella* PJI. PJIs due to *Salmonella* remain rare, and the presentation is often acute with fever. It frequently occurs in immunocompromised patients. In our patient population, removal of prosthesis with or without reimplantation, along with 4–6 weeks of effective parenteral antimicrobial therapy was most often associated with successful eradication of infection.

## Background

By 2030, the annual number of combined knee and hip arthroplasties is estimated to reach four million in the United States [1]. Infections following joint prosthesis implantation occur at a frequency of 1-3%, and are a major cause of morbidity and healthcare expenditure [2],[3].

Staphylococci are the most common identified organism in prosthetic joint infections (PJIs). *Salmonella* are an enteroinvasive Gram- negative organism with capacity to cause bacteremia and seeding of various organs. Despite their ability to cause native bone and joint infections, especially in immunocompromised individuals [4],[5], *Salmonella* PJIs occur rarely. There have been numerous multi-state outbreaks of *Salmonella* gastroenteritis throughout the United States in the last decade despite regulatory measures [6]-[8]. Some of these outbreaks involve multi-drug resistant organisms [9]. Increased global travel has also predisposed individuals to acquire infection in areas of high *Salmonella* carriage rate.

While rates of *Salmonella* gastroenteritis have increased over the past decade, there have been only infrequent case reports of *Salmonella* PJIs in this time period [10]-[12]. Being a reportable disease, one can assume high levels of detection of *Salmonella* infection, especially PJI. The ideal management regimens and outcomes of these infections are also not well defined.

We therefore retrospectively reviewed all patients with a diagnosis of PJI caused by *Salmonella* seen at our institution between 1969–2013, and evaluated the demographics, management, and outcomes of these infections.

## Methods

### Study design

This is a single center retrospective case series undertaken at the Mayo Clinic, Rochester. The study was approved by our Institutional Review Board (IRB # 14–001299, 03/07/2014). Medical and surgical therapies were not standardized and were performed at the discretion of the treating physicians.

### Study population and case ascertainment

Study patients were evaluated at our institution between 1/1/1969 and 12/31/2013. Cases were ascertained by searching our institution’s medical and surgical indices, and the microbiology database. Patients over 18 years of age that met our case definitions were included. Detailed information was abstracted from the medical records using a standardized data collection tool. Information was available for all patients. Patients were followed until the development of treatment failure, death or loss to follow- up. Descriptive statistics were used to summarize the demographic, clinical and treatment details and were analyzed using JMP, Version 9.0.1 (SAS Institute Inc.).

### Definitions

*Salmonella* PJI was diagnosed if at least one of the following criteria was met: isolation of *Salmonella* species from two cultures of joint aspirates or intraoperative tissue specimens, purulence surrounding the prosthesis at the time of surgery with one positive joint culture yielding *Salmonella* species, acute inflammation consistent with infection on histopathological examination of periprosthetic tissue with one positive joint culture yielding *Salmonella* species, or sinus tract communicating with the prosthesis with one positive joint culture yielding *Salmonella* species.

Patients were either classified as having a good outcome or having failed treatment. Treatment failure was defined by one of the following criteria: recurrence of PJI due to the same *Salmonella* strain or a different microorganism; death due to prosthesis-related infection and indeterminate clinical failure, defined as clinical, laboratory, or radiological findings suggestive of PJI at any time after initial therapy. Patients who did not fulfill criteria for treatment failure were characterized as having a ‘good outcome’.

Ethical Review Statement: IRB# 14–001299 (03/07/2014) of the Mayo Clinic, Rochester.

## Results

### Patient cohort

In our cohort of six patients, median age at diagnosis of first episode of *Salmonella* PJI was 63.5 years (range 52–76). Five patients were male. Three patients were diagnosed prior to 1984. A summary of the six patients is given in Table 1.

**Table 1:** **Summary of the 6 patients with 10 episodes of** ***Salmonella*** **Prosthetic joint infections** ^**1**^

Patient no.	Prosthetic joint (indication)	Co-morbidities	Prosthesis age at time of infection	Clinical presentation	Microbiology	Antimicrobial therapy	Surgical management	Failed treatment	Follow-up duration
1.	Left TKA (RA)	RA, Bladder cancer	3 years	Deep joint pain, discomfort x 4 months	Salmonella enterica serovar Enteritidis (aspirate)	Oral TMP-SMX x 12 weeks	Aspiration only	Yes	3 months
	‘’	‘’	3 years	Pain in region of joint, unresolved after 3 months of suppressive oral therapy	‘’ (operativesample)	Oral TMP-SMX x 8 weeks	Removal, debridement and arthrodesis	No	15 years
2.	Right THA (DJD)	Colonic polyps status post colectomy	6 years	Cholecystectom y 2 weeks prior, with positive blood and stool cultures. Fever and joint pain x 5 days	Salmonella enterica serovar Enteritidis (operative sample)	IV ampicillin x 4 weeks and oral amoxicillin x 10 months	Debride and retain	Yes	11 months
	‘’	‘’	7 years	Pain in region of prosthetic joint, unresolved after 11 months of suppressive oral therapy	‘’ (operative sample)	IV ampicillin x 4 weeks	Removal, debridement and arthrodesis	No	8 years
3	Left THA (dislocated hip)	Diabetes mellitus	5 months	Fever, joint pain, joint swelling starting 5 day prior, negative stool cultures but diarrhea 1 week earlier	Salmonella enterica serovar Choleraesui s (aspirate)	Oral ampicillin x 8 weeks	Aspiration only	Yes	2 months
	‘’	‘’	7 months	Continuing symptoms	‘’ (operative sample)	Oral ampicillin x 4 weeks	Two- stage exchange	No	21 years
4.	Left THA (DJD)	CLL, multiple skin cancers	4 years	Fever, chills x 2 days, Joint pain and swelling x 2 days	Salmonella enterica serovar Enteritidis (operative sample)	Oral ciprofloxacin x 8 weeks	Two- stage exchange	No	10 years
5.	Left THA (aseptic necrosis)	Ulcerative colitis, now on 6-MP	5 months	Fever and joint pain x 2 weeks	Salmonella enterica serovar Enteritidis (operative sample)	IV ceftriaxone x 6 weeks	Two- stage exchange	No	12 years
6.	Left THA (DJD)	CKD, h/o stroke	9 years	Complicated Salmonella UTI and septic shock 1 month prior, now left hip joint pain x 4 days	Salmonella bongori (aspirate)	Oral ciprofloxacin x 8 weeks	Aspiration only	Yes	2 months
	‘’	‘’	9 years	Continuing hip pain	‘’ + MSCONS, viridans group Streptococc us sp., (operative sample)	IV ceftriaxone x 6 weeks	Two- stage exchange	No	4 years

^1^M- male, F- female, TKA- total knee arthroplasty, THA- total hip arthroplasty, RA- rheumatoid arthritis, TMP-SMX – trimethoprim-sulfamethoxazole, DJD- degenerative joint disease, CLL- chronic lymphocytic leukemia, 6-MP- 6-mercaptopurine, CKD- chronic kidney disease, UTI- urinary tract infection, MSCONS- methicillin- susceptible Coagulase negative *Staphylococci.*

Five patients had a total hip arthroplasty (THA) infection while one patient had total knee arthroplasty (TKA) infection. Indication for joint arthroplasty included degenerative joint disease (3), and traumatic femoral head dislocation, rheumatoid arthritis and steroid induced avascular femoral head necrosis (1 each). Median age of prosthesis at time of diagnosis of first episode of *Salmonella* PJI was five years (range 4 months-9 years). No patient had history of prior PJI on the same or different joint.

Five patients were immunosuppressed. One patient each had diabetes mellitus, chronic kidney disease, rheumatoid arthritis (RA), chronic lymphocytic leukemia (CLL) and ulcerative colitis taking 6-mercaptopurine (6-MP).

Five patients presented acutely with signs and symptoms present for less than two weeks. Median duration of symptoms prior to diagnosis was 4.5 days (range 2 days-4 months). Only one patient, a 52 year-old-male with rheumatoid arthritis on steroids, presented with chronic symptoms (4 months of deep hip pain). All patients presented with local pain around the prosthesis. Four patients were febrile at presentation. None of the patients had an endovascular infection. None of the patients presented with or developed a discharge sinus during follow- up.

Two patients had diarrhea in the preceding two weeks before symptom onset, of which one had positive stool *Salmonella* culture, and two patients had documented Salmonella bacteremia before the PJI was diagnosed.

### Diagnosis

All first episodes of *Salmonella* PJI were diagnosed in accordance with the definitions described in the methodology section. None of the first episodes had co-infection with another organism. *Salmonella enterica* serovar Enteritidis was the most common organism (4 patients) with *S. enterica* serovar Choleraesuis and *S. bongori* causing one infection each. All isolates were pan-susceptible with one being non-susceptible to nalidixic acid but susceptible to ciprofloxacin.

Median erythrocyte sedimentation rate (ESR) and C - reactive protein (CRP) values at presentation were 84 mm/1^st^ hour (range 46–147), and 3.2 mg/ dL (range 1.4- 8.3) respectively (normal range of ESR 0–22 mm/ 1^st^ hour, and CRP <8 mg/dL). Three patients had available pathology reports; two had acute inflammation while one had fibroreactive changes without evidence of acute inflammation.

### Management and outcome

Four patients were initially managed without removal of prosthesis, of which three were diagnosed prior to 1984 and one patient diagnosed later initially declined surgical intervention. Three patients were initially managed with oral antimicrobial therapy alone (with diagnostic aspiration) for a median duration of 8 weeks (range 8–12), while one patient underwent debridement and retention of prosthesis along with four weeks of parenteral antimicrobial therapy and ten months of suppressive oral therapy. Three of these four patients were diagnosed prior to 1984 while one patient was diagnosed in 2010 (declined surgical intervention initially). The two patients, diagnosed in 1994 and 2002, that underwent a two-stage exchange as primary management were successfully treated.

Median time to treatment failure for the four patients managed non-operatively or with debridement and component retention was 2.5 months (range 2–11). They all presented with incompletely resolved pain in the region of the prosthesis. Operative cultures identified the same organism as causing first PJI in all four patients, with one patient also growing methicillin- susceptible coagulase-negative *Staphylococcus* and viridans group *Streptococcus* species*.* Definite surgical therapy was later performed on all four patients, with two patients managed with implant removal and arthrodesis, and two with two- stage exchange.

In total, all six cases required removal of prosthesis for cure including four after failure of medical therapy alone. Median duration of follow- up after prosthesis removal was 11 years (range 4–21). Patients who underwent a two-stage exchange had a resection arthroplasty with placement of antibiotic (vancomycin and gentamicin) impregnated bone cement followed by delayed prosthesis re-implantation (6–8 weeks after prosthesis removal).

Three of the six patients managed with prosthesis removal received oral antimicrobial therapy (1 each trimethoprim-sulfamethoxazole, ampicillin and ciprofloxacin), median duration 8 weeks (range 4–8). Another three received parenteral antimicrobial therapy, median duration 6 weeks (range 4–6).

## Discussion

*Salmonella* PJIs are rare and only 29 cases were identified in a recent literature review published in 2012 [13]. They were responsible for 0.2% of all PJIs at our institute over the study period. *Salmonella* illness remains a major public health problem in the United States, with an estimated 1.4 million human *Salmonella* infections, 15,000 hospitalizations, and 400 deaths annually [14]. There have been several multi-state outbreaks of *Salmonella* throughout the United States in the last decade despite regulatory measures, usually related to contaminated or inadequately processed food products and animal exposure [6]-[9]. This tremendous spike in *Salmonella* infections has not coincided with an increase in *Salmonella* PJI cases seen at a single center or reported in the literature. We did not see an increase in *Salmonella* PJI cases at our institute either. Perhaps these outbreak associated infections are less prone to causing PJI.

*Salmonella* are acquired through the feco-oral route or through animals or food. Almost half the patients with *Salmonella* PJI have gastroenteritis prior to diagnosis [15]. Two of six patients in our cohort had diarrhea prior to diagnosis of PJI with one having positive stool *Salmonella* culture. Initial gastrointestinal salmonellosis can be mild and may go unnoticed. The upsurge in food-borne illnesses in the United States [16] and recent reports highlighting PJIs caused by *Campylobacter* [17]*, Yersinia* [18]*,* and *Clostridium difficile* [19],[20] *suggest* that patients and clinicians should be alert to diarrheal episodes in patients with prosthetic joints.

The route of *Salmonella* in causing PJI is generally considered to be hematogenous [21],[22]. *Salmonella* can seed various organs, and their ability to cause native bone and joint infections is well recognized, especially in patients with sickle cell disease [22]. The time between implantation and infection can provide a clue about the source of infection with early and delayed PJI (joint age less than one year) more likely to be intra-operatively acquired. The literature shows varied time to infection with a range from 3 days - 14 years [13]. In our cohort, the median prosthesis age at infection was five years. Majority were THA, the significance of which is not clear. The detection of confirmed *Salmonella* bacteremia in two patients before the diagnosis of PJI (with the same species identified in operative cultures) supports hematogenous dissemination as the pathway for infection. Romero, et al. documented three episodes of *Salmonella* PJI in renal transplant recipients, two of which had a proven *Salmonella* urinary tract infection (UTI) prior to diagnosis [23], similar to one of our patients who had UTI and bacteremia a month prior to diagnosis of PJI.

The three patients in our cohort who did not have positive blood cultures or diarrhea prior to PJI were immunocompromised- one had CLL, other was taking steroids for RA, and the last one took 6-MP for ulcerative colitis. Chun, et al. have suggested that ‘gut bacterial translocation’ may be a portal of entry of *Salmonella* into the bloodstream, especially in immunodeficient individuals, as demonstrated in a patient with rheumatoid arthritis taking steroids who developed *Salmonella* PJI [24].

Non-typhoid *Salmonella* have traditionally caused PJIs more commonly than typhoidal *Salmonella* [13]*,* with very few cases of the latter reported [25]. *Salmonella enterica* serovar Enteritidis was the most commonly isolated organism in our cohort.

There have been reports of multi-drug resistant *Salmonella* strains causing bone and joint infections [22],[26]. All the isolates in our cohort were pan-susceptible with a single isolate showing in-vitro resistance to nalidixic acid. Fluoroquinolones are widely advocated as excellent first choice drugs for *Salmonella* PJIs given their high oral bio-availability and ability to penetrate biofilms and kill stationary phase as well as active phase bacteria. Widmer, et al. demonstrated their efficacy over trimethoprim-sulfamethoxazole (TMP-SMX) in curing *Salmonella* PJI and provided an explanation involving biofilms using a foreign body animal model [27]. Treatment failure has been reported with TMP-SMX use. [28] One patient in our cohort was treated with oral TMP-SMX initially. Although this patient failed treatment with oral TMP-SMX alone, infection was cleared with removal of the prosthesis and continuing oral TMP-SMX later.

PJI is primarily a surgical disease and most patients require surgical intervention for management. Although sporadic reports of successful management of *Salmonella* PJI have been reported with implant retention [12],[29], other series have supported the use of prosthesis removal for successful outcome [13],[21],[30]. Based on current management consensus, patients with PJI symptoms less than 4 weeks can be treated with debridement and prosthesis retention. In patients presenting with discharge sinus or with symptoms greater than four weeks, two-stage exchange arthroplasty is indicated. Debridement alone had a high failure rate in our study despite acute onset of symptoms (median duration of symptoms was 4.5 days), and the same has been reported in patients with PJI caused by other Gram-negative organisms especially when the duration of symptoms is long [31]. In our cohort, all four patients who failed treatment were initially managed with implant retention and had successful outcomes with prosthesis removal. One patient in our cohort refused any surgical intervention initially (patient 4) and was hence given eight weeks of oral ciprofloxacin. When this did not result in resolution of symptoms, he allowed surgical intervention and was treated successfully using two-stage exchange and six weeks of parenteral ceftriaxone.

Reported success rates described in the literature may differ owing to the limited duration of follow-up, difference in the definition of a successful outcome, and the propensity for publication bias of successfully treated case reports. In our cohort, the median duration of follow up was eleven years. The major limitations to our study are inherent to its observational retrospective nature. The small sample size is a function of the low incidence of the disease. Since our data was collected from a single tertiary referral medical center, there is a potential for a referral bias. Management techniques have evolved over the long study period of more than forty years. Nevertheless, this series of *Salmonella* PJIs supports the use of prosthesis removal in these patients.

## Conclusions

The increase in *Salmonella* outbreaks does not seem to be leading to increased *Salmonella* PJI. *Salmonella* remains a rare but well recognized cause of PJI. *Salmonella* PJI should be suspected in patients with history of diarrheal illness, documented *Salmonella* infection elsewhere in the body, or in immunocompromised patients. Presentation is often acute with fever and local signs, and elevated ESR and CRP levels. Treatment requires a combined medical and surgical approach. Prosthesis removal is associated with higher chance of cure compared to implant retention. Both oral and parenteral antibiotics have been used for medical therapy guided by antimicrobial susceptibilities and patient characteristics.
